# Multifractality and Network Analysis of Phase Transition

**DOI:** 10.1371/journal.pone.0170467

**Published:** 2017-01-20

**Authors:** Longfeng Zhao, Wei Li, Chunbin Yang, Jihui Han, Zhu Su, Yijiang Zou

**Affiliations:** 1 Complexity Science Center & Institute of Particle Physics, Hua-Zhong (Central China) Normal University, Wuhan 430079, China; 2 Max-Planck-Institute for Mathematics in the Sciences, 04103 Leipzig, Germany; Technical University of Madrid, SPAIN

## Abstract

Many models and real complex systems possess critical thresholds at which the systems shift dramatically from one sate to another. The discovery of early-warnings in the vicinity of critical points are of great importance to estimate how far the systems are away from the critical states. Multifractal Detrended Fluctuation analysis (MF-DFA) and visibility graph method have been employed to investigate the multifractal and geometrical properties of the magnetization time series of the two-dimensional Ising model. Multifractality of the time series near the critical point has been uncovered from the generalized Hurst exponents and singularity spectrum. Both long-term correlation and broad probability density function are identified to be the sources of multifractality. Heterogeneous nature of the networks constructed from magnetization time series have validated the fractal properties. Evolution of the topological quantities of the visibility graph, along with the variation of multifractality, serve as new early-warnings of phase transition. Those methods and results may provide new insights about the analysis of phase transition problems and can be used as early-warnings for a variety of complex systems.

## Introduction

Complex systems are formed by subunits that interact non-linearly with each other. Among so many general properties that describe the complex systems, the existence of critical threshold is apparently common to plenty of complex systems [1]. There are many examples of critical transitions that pose potential threats to our daily life. Such potentially dangerous examples include spontaneous systemic failures disease for human beings, systemic market crashes for global finance, abrupt shifts in ocean circulation or climate and so on. It is not possible, for those complex systems, to fully anticipate their behaviors in terms of behaviors of their components. Thus characterizing the dynamical process of complex systems from macroscopic quantity, for example time series, is a fundamental problem of significant importance in many research fields [2, 3].

Recently there has been an substantial interest in understanding how those complex systems behave near the critical point. Hence lots of early-warnings have been proposed to serve as signals of the coming of the tipping points [1]. Thereinto, the early-warnings based on time series analysis are fairly common. It is well known that fractal and multifractal time series are ubiquitous in numerous complex systems [4]. Lots of techniques have been proposed to analyze the fractal and multifractal properties of time series [5–11]. Multifractal detrended fluctuation analysis (MF-DFA) [7] has shown to be quite effective to investigate the multifractal properties of non-stationary time series. Furthermore, the complex network [12] is one of the generic ways to describe complex systems. Transformations from time series to networks have attracted substantial considerations recently [2, 13–16]. Among so many techniques, the visibility graph method [14] is suitable for characterizing the geometrical structure of time series [17]. Thus utilizing the MF-DFA and visibility graph method to inspect the critical behaviors of complex systems is an interesting topic.

Here we focus on the well-known physical system, the two-dimensional Ising model [18, 19]. It is a statistical physics model for ferromagnetic materials which goes through spontaneous phase transition at non-zero temperature. The Ising model has been widely applied to different fields such as social systems [20] and financial systems [21]. We use the Metropolis algorithm [22]to simulate the two-dimensional Ising model. The outputs of the simulations are time series of average magnetization at different temperatures. The time evolution of average magnetization can be used to reveal the dynamical properties of the system. We then take advantage of the MF-DFA and the visibility graph method to analyze the behavior of the Ising model near critical temperature. Hurst exponents increase dramatically around critical point which means the time series change from short correlated to long-term correlated ones. Generalized Hurst exponents have shown the transformation of fractal structures of the time series from weak multifractal (or monofractal) to strong multifractal while the system approaches critical point. Evolution of the singularity spectrum has depicted the extremely strong multifractality around the critical point. Structural parameters of the singularity spectrum suggest that the time series become much more complex around the critical point. Thus huge differences between the dynamical behaviours of the Ising model at different temperatures in the time domain have been uncovered. The shuffling procedure reveals that both broad probability density function and long-term correlations are the sources of multifractality around critical point. Visibility graphs converted from the time series at different temperatures share the heterogeneous nature. We also find that the increase and decrease of the topological quantities can be used to identify the coming of phase transition. The evolution of topological structures have manifested the differences among geometrical structures of magnetization time series at different temperatures from the complex network perspective. In summary, the multifractality of the time series and topological quantities of the complex networks converted from the time series can be seen as some new metric-based early-warnings.

## Methods

### Simulation of the Ising model

The two-dimensional Ising model is a paradigm of physical phase transition. Suppose we have a square lattice of *N* sites with periodic boundary conditions. A spin state *σ* is defined on each site with one of two possible orientation values denoted by *σ* = ±1. So the number of all possible configurations of the system is 2^*N*^. The Hamiltonian is

$$\begin{array}{c} H\left(\sigma \right)=-\sum_{<i,j>}J_{ij}\sigma_{i}\sigma_{j}-\mu \sum_{i=1}^{N}h_{i}\sigma_{i}. \end{array}$$ (1)

Any two adjacent sites *i*, *j* ∈ *N* have an interaction strength *J*_*ij*_. A site *i* ∈ *N* has an external magnetic field *h*_*i*_ acting on it and *μ* is the magnetic moment. The order of the system can be measured through the magnetization per spin which is defined as

$$\begin{array}{c} M=\frac{1}{N}\sum_{i=1}^{N}\sigma_{i}. \end{array}$$ (2)

It is well known that for the two-dimensional Ising model the system goes through a phase transition when temperature *T* equals to the critical temperature *T*_*c*_. There exists spontaneous magnetization as all the spins tend to equal toward either +1 state or −1 state at a non-zero critical temperature *T*_*c*_.

Here in this paper we focus on the dynamical evolution of the Ising model. The Metropolis algorithm [22] is employed to simulate the model. Thus the time series of magnetization *M* can be obtained from simulation procedure.

In the Metropolis algorithm, new configurations are generated from the previous states via a transition probability. The probability of the system being in a state *n* follows the Boltzmann distribution:

$$\begin{array}{c} P_{n}=\frac{e^{-E_{n}/kT}}{Z}, \end{array}$$ (3)

where *E*_*n*_ is the free energy of the state, *k* is the Boltzmann constant, *T* is the temperature and *Z* is the partition function. Thus the transition probability from state *n* to *m* is given by

$$\begin{array}{c} P_{n\to m}=\text{exp}\left[-\Delta E/kT\right], \end{array}$$ (4)

where Δ*E* = *E*_*m*_ − *E*_*n*_.

The algorithm proceeds as follows:

1. Choose a site *i* randomly;
2. Calculate the energy change Δ*E* if spin site *i* were to be flipped;
3. If Δ*E* is negative, flip of the spin of site *i* is accepted. If Δ*E* is positive, a random number is drawn from a uniform distribution between 0 and 1 and the flip is accepted only if the random number is smaller than exp[−Δ*E*/*kT*];
4. Choose another site and repeat the previous steps.

A Monte Carlo step is completed when every spin of the system has had a chance to flip. In the ordinary simulation scenario, due to the phenomena of critical slowing down, when the system approaches the critical temperature *T*_*c*_, several Monte Carlo steps should be skipped in order to avoid correlations between successive configurations. This is important for evaluating the quantities of interest accurately. On the contrary, here we keep all the time series of magnetization *M* calculated from every simulation step. We are indeed very interested in the correlations and the properties caused by the critical slowing down phenomena.

In the following simulation, *k* and *J*_*ij*_ are set to be 1. The critical temperature is *T*_*c*_ ≃ 2.27. We have simulated the system from *T* = 1.17 to *T* = 3.62 with Δ*T* = 0.05. For every discrete temperature we run an ensemble of 100 simulations of 100,000 Monte Carlo steps (the first 10,000 steps have been discarded to overcome the influence of the initial configuration). In order to investigate the finite size effect, we have simulated different lattice sizes for *N* = 100 × 100, 150 × 150, 200 × 200 and 300 × 300. Then in the following section we will introduce two methods that will be used to analyze the properties of those time series from two different aspects: multifractal and complex network.

### Multifractal Detrended Fluctuation Analysis

We adopt multifractal detrended fluctuation analysis (MF-DFA) to analyze the hierarchy of scaling exponents of the magnetization time series corresponding to different scaling behaviour [7]. The MF-DFA method is the generalization of detrended fluctuation analysis (DFA) [5] and has been widely applied to characterize the properties of various non-stationary time series in different fields such as financial market [23–32], physiology [33], biology [34], traffic jamming [35], geophysics [36] and neuroscience [37].

The MF-DFA method proceeds as follows: (*i*) Suppose we have a time series {*x*(*i*)}, *i* = 1, …, *l*. We first integrate the time series to generate the profile $y\left(k\right)=\sum_{i=1}^{k}\left[x\left(i\right)-\langle x\rangle \right],k=1,\ldots ,l$, where 〈*x*〉 is the mean value of {*x*(*i*)}. (*ii*) Divide the integrated series *y*(*k*) into *l*_*s*_ = *int*(*l*/*s*) non-overlapping segments of length *s*. Calculate the local trends for each of *l*_*s*_ segments by a least-square fit and subtract it from *y*(*k*) to detrend the integrated series. We then obtain the detrended variance of each segment *v*

$$\begin{array}{c} F^{2}\left(v,s\right)=\frac{1}{s}\sum_{i=1}^{s}{\left\{y\left(v-1+i\right)-\tilde{y(v,i)}\right\}}^{2}, \end{array}$$ (5)

where $\tilde{y(v,i)}$ is the fitted trend in segment *v* = 1, …, *l*_*s*_. We use a third order polynomial to fit the local trend here. (*iii*) Step (*ii*) has been proceeded from both the beginning and the end of the time series which leads to 2*l*_*s*_ segments in total. Average over all segments to obtain the *qth* order fluctuation function

$$\begin{array}{c} F_{q}\left(s\right)={\left\{\frac{1}{2l_{s}}\sum_{v=1}^{2l_{s}}{\left[F^{2}\left(v,s\right)\right]}^{q/2}\right\}}^{1/q}, \end{array}$$ (6)

with *q* a real number, and for positive *q*, *F*_*q*_(*s*) measures large fluctuations, while for negative *q*, *F*_*q*_(*s*) measures small fluctuations. (*iv*) Repeat this calculation to get the fluctuation function *F*_*q*_(*s*) for different box size *s*. If *F*_*q*_(*s*) increases by a power law *F*_*q*_(*s*)∼*s*^*h*(*q*)^, then the scaling exponents *h*(*q*) (called generalized Hurst exponents) can be estimated as the slope of the linear regression of *logF*_*q*_(*s*) versus *log*(*s*). *h*(*q*) are the fluctuation parameters and describe the correlation structures of the time series at different magnitudes. The value of *h*(0) can not be determined by using Eq (6) because of the diverging exponent. The logarithm averaging procedure should be used,

$$\begin{array}{c} F_{0}\left(s\right)=\text{exp}\left\{\frac{1}{4l_{s}}\sum_{v=1}^{2l_{s}}\text{ln}\left[F^{2}\left(v,s\right)\right]\right\}∼s^{h(0)}. \end{array}$$ (7)

The generalized Hurst exponents *h*(*q*) as a function of *q* can quantify the multifractality. If *h*(*q*) are the same for all *q*, the time series is monofractal, otherwise the time series is multifractal.

The classical multifractal scaling exponents *τ* defined by the standard partition function-based multifractal formalism are directly related to the generalized Hurst exponents *h*(*q*): [38]

$$\begin{array}{c} \tau (q)=qh(q)-1. \end{array}$$ (8)

Another way to characterize multifractal time series is by using the singularity spectrum *f*(*α*) which is related to *τ*(*q*) via the Legendre transform [7],

$$\begin{array}{c} \alpha =\tau^{{}^{\prime }}\left(q\right),\;f\left(\alpha \right)=q\alpha -\tau \left(q\right). \end{array}$$ (9)

Using Eq (8), we have

$$\begin{array}{c} \alpha =h\left(q\right)+qh^{{}^{\prime }}\left(q\right),\;f\left(\alpha \right)=q\left[\alpha -h\left(q\right)\right]+1. \end{array}$$ (10)

Here *α* is the singularity strength. Geometrical shape of the singularity spectrum can illustrate the level of multifractality with three parameters: position of the maximum *α*_0_ where *f*(*α*) reaches its maxima; width of the spectrum *W* = *α*_*max*_ − *α*_*min*_ which can be obtained from extrapolating the fitted *f*(*α*) curve to zero; and skew shape of the spectrum *r* = (*α*_*max*_ − *α*_0_)/(*α*_0_ − *α*_*min*_). In a nutshell, large value of *α*_0_ means the underlying process is more irregular. The wider the spectrum (larger *W*), the richer the structure of the time series. The skew parameter *r* determines which fractal exponents are dominant. Right skew shape of the spectrum with *r* > 1 is more complex than left skew shape with *r* < 1 [31, 39].

### Visibility Graph Method

Complex network and time series are two generic ways to describe complex systems. Geometrical properties of time series can usually be preserved in network topological structures. Lots of methods have been developed to capture the geometrical structure of time series from complex network aspect such as cycle network [2], correlation network [40], visibility graph [14], recurrence network [41], isometric network [15] and many others. Among those methods, the visibility graph has a straight forward geometric interpretation of the original time series. Namely, periodic time series can be transformed into regular networks and random series corresponding to random networks [42]. Moreover, fractal series can be converted into scale-free networks [43]. Hence the visibility graph method has been successfully applied to many fields [35, 44, 45]

The visibility graph algorithm can be described as follows: for a time series {*x*(*i*)}, *i* = 1, …, *l*, two arbitrary data points (*t*_*i*_, *x*(*i*)) and (*t*_*j*_, *x*(*j*)) will have visibility, and those two data points will become two connected nodes *i* and *j* of the associated network connected by edge *e*_*ij*_, if all the data points (*t*_*k*_, *x*(*k*)) placed between (*t*_*i*_, *x*(*i*)) and (*t*_*j*_, *x*(*j*)) fulfill:

$$\begin{array}{c} x\left(k\right)<x\left(j\right)+\left(x\left(i\right)-x\left(j\right)\right)\frac{t_{j}-t_{k}}{t_{j}-t_{i}}. \end{array}$$ (11)

The network obtained from this algorithm will always be connected. The network is also undirected because we do not keep any direction information in the transformation.

## Results and Discussion

### Statistical Properties of Time Series

**Fig 1:**
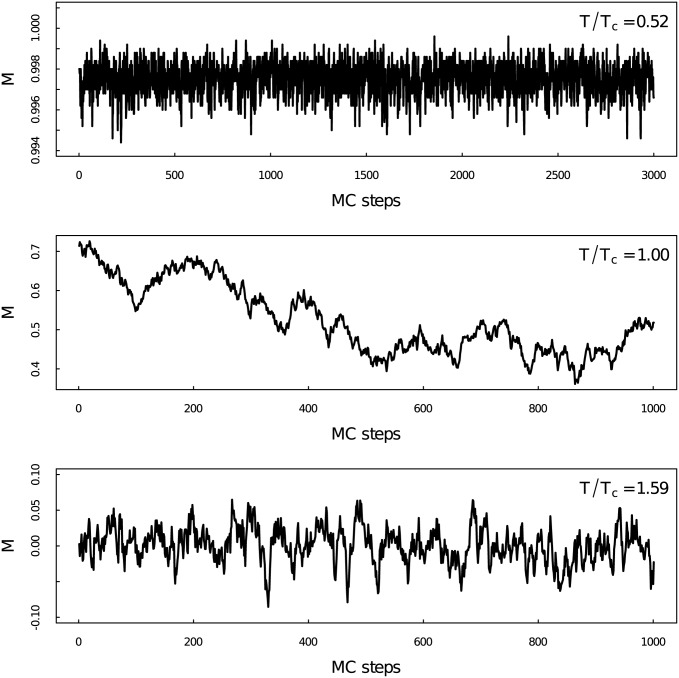
Average Magnetization time series per spin. Average magnetization *M* as a function of time for the two-dimensional Ising model at different temperatures.

Fig 1 shows the time series at three temperatures *T*/*T*_*c*_ = 0.52, *T*/*T*_*c*_ = 1.00, and *T*/*T*_*c*_ = 1.59 from top to bottom. Obviously we can observe different outlines of the time series at different temperature regions. Detailed properties of those time series will be analyzed by using multifractal detrended analysis and visibility graph method in the following context.

**Fig 2:**
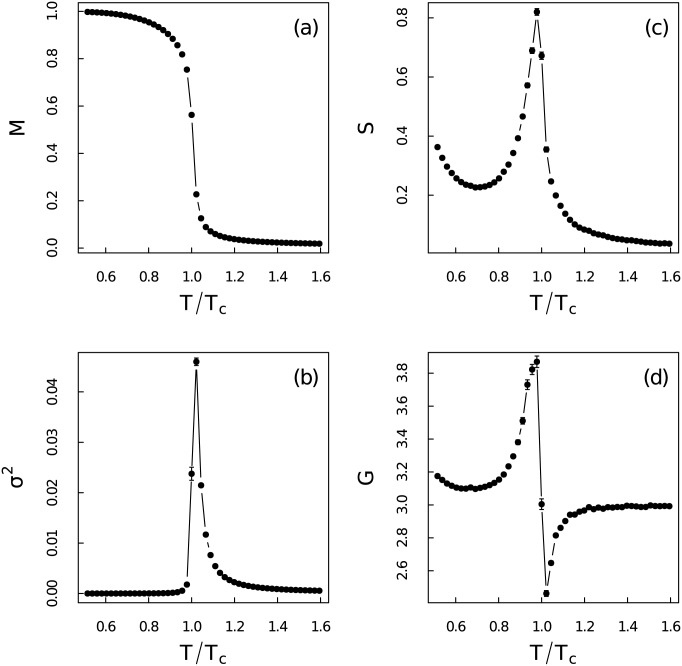
First four moments of the magnetization time series. Ensemble average of (a) mean *M*, (b) variance *σ*^2^, (c) skewness *S* (d) kurtosis *G* of the magnetization time series versus the relative temperature *T*/*T*_*c*_ for system size *N* = 100 × 100.

The first four order moments are used to quantify the distribution differences between those time series in Fig 2. The ensemble standard errors are defined as standard deviation divided by square root of the ensemble size (100) and are shown as error bars in figures. Fig 2(a) is the ensemble average magnetization *M* varying with relative temperature *T*/*T*_*c*_. Simulation results have confirmed the existence of phase transition around the theoretical critical temperature *T*_*c*_ ≃ 2.27. This transition can also be verified from three statistical quantities in Fig 2: (b) variance *σ*^2^, (c) skewness *S* and (d) kurtosis *G* which have been fully discussed in Ref [46]. Those moments have been used as early-warnings of phase transition therein. The variance *σ*^2^ is almost zero at non-critical regions, but it becomes relatively large around critical point. The abrupt change at low temperature region is obviously different from the continuous evolution at high temperature region. Thus the variance can not be recognized as a useful early-warning when the system approaches the critical point from low temperature region. The skewness *S* is related to the asymmetry of events in the time series. It is large only in the low temperature region and reaches its maxima at critical point and then becomes very small at high temperature region. This is due to the meta-stable states at low temperature [46]. The stochastic permutation is not strong enough to make the system escape from the meta-stable states. This makes the distribution of magnetization asymmetric. The kurtosis *G* fluctuates severely around critical point and becomes very close to the reference normal distribution (which has a kurtosis equals to 3) for *T* ≪ *T*_*c*_ and *T* ≫ *T*_*c*_. Apparent distribution differences between magnetization time series at different temperature regions give a hint about the structure heterogeneity which demands more detailed investigations.

### MF-DFA

**Fig 3:**
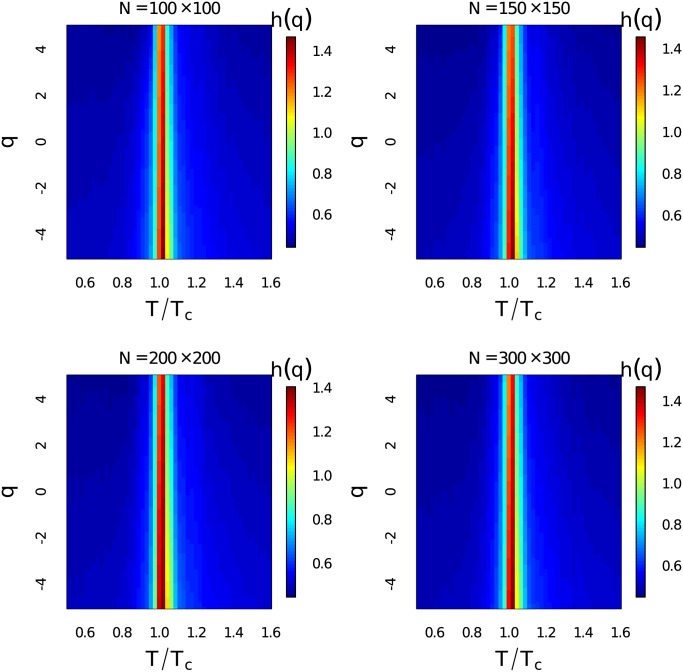
Heat map of the generalized Hurst exponent. (Color online) Heat map of the ensemble average of the generalized Hurst exponent *h*(*q*) for *q* ∈ [−5, 5] at different temperatures with different system sizes.

The generalized Hurst exponents *h*(*q*) at different temperatures for *q* ∈ [−5, 5] with Δ_*q*_ = 0.1 have been demonstrated in Fig 3. The system sizes are *N* = 100 × 100, 150 × 150, 200 × 200 and 300 × 300. The time series length here for MF-DFA is 100,000. Heat map of *h*(*q*) has given a comprehensive description about scaling behaviours of the fluctuations at different magnitudes. Dramatic increase of the generalized Hurst exponent around critical temperature at different order *q* can be observed. The generalized Hurst exponent is very close to 0.5 and then it becomes remarkably larger than 1 around critical point at all observed order *q* ∈ [−5, 5]. This indicates that the generalized Hurst exponent is a very good indicator of phase transition.

**Fig 4:**
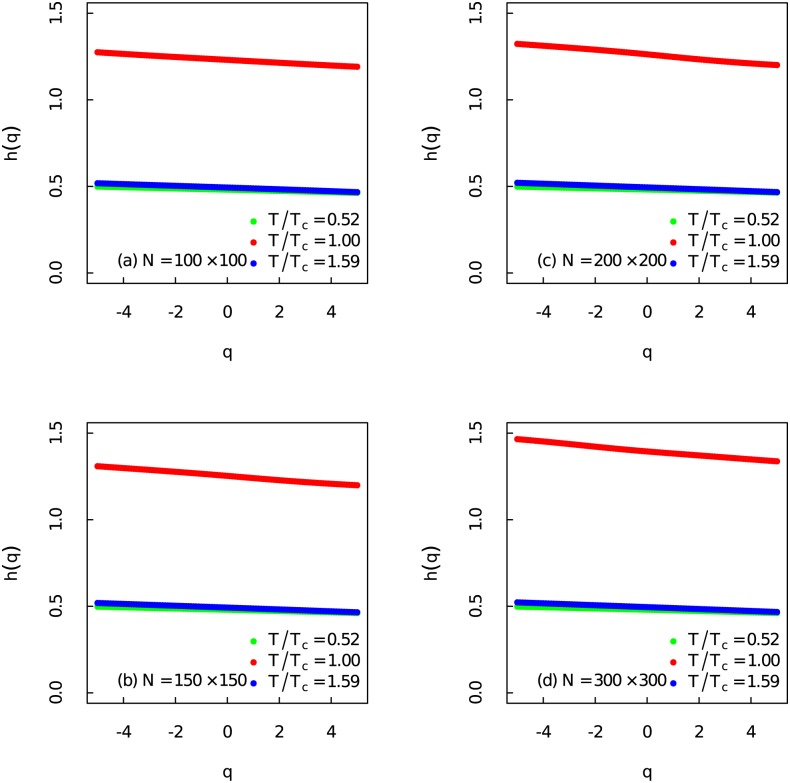
Generalized Hurst exponent at three different temperatures. (Color online) The generalized Hurst exponent *h*(*q*) as a function of *q* at three different temperatures for different system sizes.

As presented in Fig 4, when *T*/*T*_*c*_ = 0.52 and *T*/*T*_*c*_ = 1.59 the weak dependence on *q* of *h*(*q*) show that the fractal properties of the fluctuations at different magnitude are almost the same, thus the time series far away from the critical point are weak multifractal. As the system approaches the critical region while *T*/*T*_*c*_ ∼ 1.00, *h*(*q*) become strongly dependent on *q*. The small and large fluctuations of the Ising model near critical point display different scaling behaviours. The strong non-linear dependence on *q* of *h*(*q*) around *T*_*c*_ reveals the strong multifractal nature of the Ising model. Transformation of the multifractal properties uncover the apparent structural and dynamical differences of the Ising model at different temperature regions.

**Fig 5:**
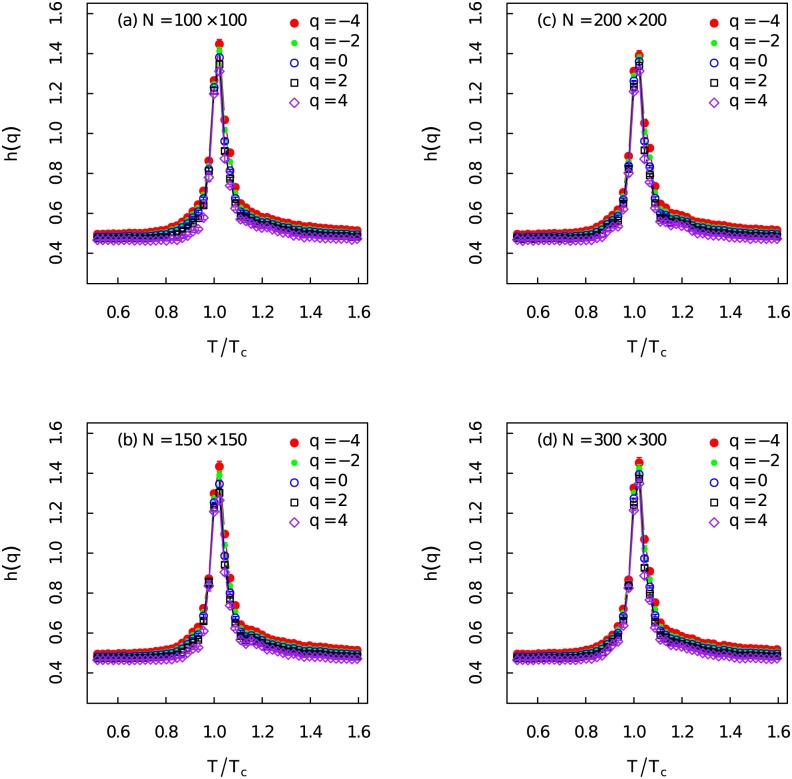
Generalized Hurst exponent for different order *q*. (Color online) Ensemble average of the generalized Hurst exponent *h*(*q*) versus the relative temperature *T*/*T*_*c*_ for different order *q* with different system sizes.

The generalized Hurst exponent for different order *q* have been displayed in Fig 5. *h*(*q*) shows the same critical behaviours for different order *q*. If we set *q* = 2 in the MF-DFA, we get the same results as the standard DFA method. The Hurst exponents *h*(*q* = 2) have been used as early-warning to detect the rising memory in time series of a system close to critical point [47, 48]. The Hurst exponent *h*(*q* = 2) ≃ 0.5 when *T*/*T*_*c*_ ≪ 1.00 and *T*/*T*_*c*_ ≫ 1.00 which means the magnetization time series are short-range correlated at these two regions. When *T*/*T*_*c*_ ∼ 1.00 the Hurst exponent increases rapidly which results in *h*(*q* = 2) > 1. It tells that the time series becomes non-stationary which turns to be an unbounded process. The Hurst exponent *h*(*q* = 2) ≥ 1.2 when the system gets very close to the critical point. This indicates that the magnetization time series posses long-range correlations. Those characteristics of the generalized Hurst exponent should be very good early-warnings [1, 46].

**Fig 6:**
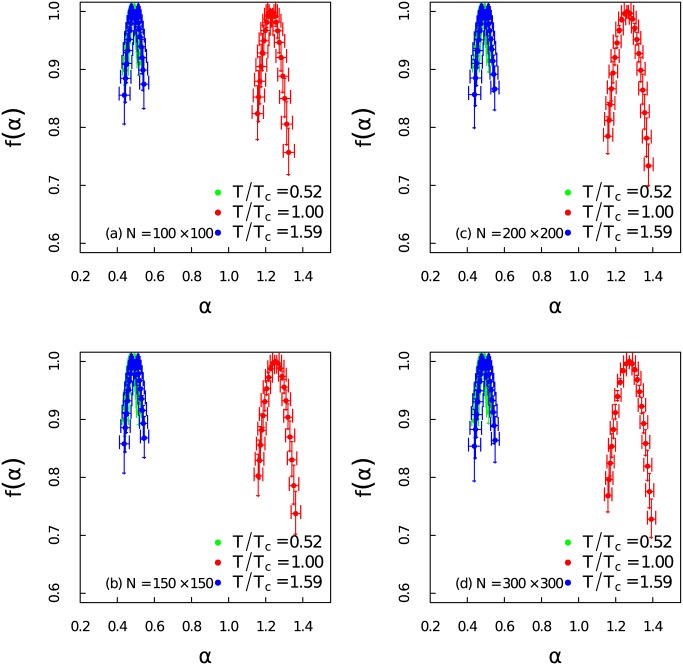
Singularity spectrum at different temperature regions. (Color online) Singularity spectrum *f*(*α*) of the time series as a function of the singularity strength *α* at different temperatures for different system sizes.

In order to quantify the level of multifracality, the singularity spectrum of the time series at different temperatures for different system sizes have been presented in Fig 6. The maximum of the *α*_0_ is 1.3 when *T*/*T*_*c*_ = 1.00, but it decreases to 0.5 when the temperature deviates from *T*_*c*_. We can observe that the width of singularity spectrum increases as *T* approaches *T*_*c*_. At the same instant the singularity becomes right skew. Those alternations indicate great complexity around critical temperature. The patterns of the singularity spectrum are basically consistent for different system sizes.

A more quantitative measure about the multifractality of the series can be given by fitting the singularity spectrum [31] and calculating the singularity spectrum parameters: position of maximum *α*_0_; width of the spectrum *W* = *α*_*max*_ − *α*_*min*_; and the skew parameter *r* = (*α*_*max*_ − *α*_0_)/(*α*_0_ − *α*_*min*_) described in the previous method section. These parameters lead to overall measures of the singularity complexity: a time series with large value of *α*_0_, a wide range *W* of fractal exponents, and a right-skewed shape may be considered more complex than the one with opposite characteristics [39].

**Fig 7:**
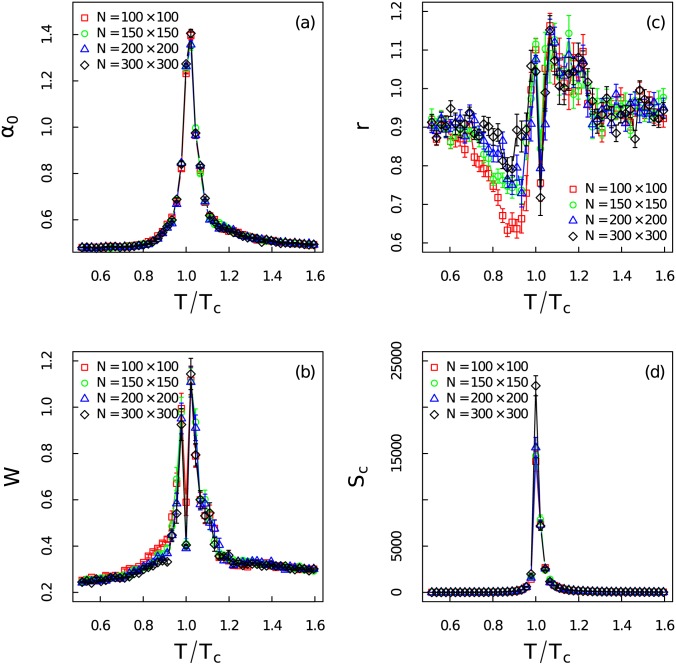
Complexity measure of the singularity spectrum and autocorrelation length of the time series. (Color online) The complexity measure of the time series at different temperatures: (a) the position of maximum *α*_0_, (b) the width of the spectrum *W*, (c) the skew parameter *r*, (d) the critical length of the autocorrelation *S*_*c*_ for different system sizes.

As shown in Fig 7(a) the value of *α*_0_ becomes very large at *T*/*T*_*c*_ = 1.00 which suggests that the time series become extremely irregular. The evolution pattern of *α*_0_ can serve as a very good early-warning about the coming of the critical transition. The increase of the width *W* of the spectrum in Fig 7(b) indicates richer structure near critical region. The abrupt jump right at the critical point gives a hint about the mutation of the correlation structure. Shrink of the width *W* has also been observed in Ref. [49] where the multifractal feature of the energy spectrum have been analyzed via the partition function approach. The skew parameters *r* in Fig 7(c) is almost equal to 1 when temperature deviates from critical region which manifests symmetry shapes of the multifractal spectrum at low and high temperature regions. It becomes larger than 1 near critical threshold. One interesting finding is that the skew parameter *r* first gradually decreases at lower temperature region and then increases very fast when the system gets particularly close to critical point. This shows that the large fluctuations dominate when the system approaches critical point from the lower temperature. On the contrary, *r* gradually reaches the maxima when the system moves close to *T*_*c*_ from the right side. Thus the small fluctuations contribute the most to the multifractality beyond the critical point. This indicates a more complex structure with right skewed shape above the critical point.

Fig 7(d) gives the critical length of the autocorrelation as a function of relative temperature. The critical length of the autocorrelation function is the maximum lag at which the autocorrelation is smaller than the critical value $2/\sqrt{l}$. According to a recent research about the DFA on autoregressive process (AR(1)), the autocorrelation length can be used to estimate the Hurst exponent more accurately [52]. It is known that the two point correlation function of the Ising model will become divergent in the thermodynamic limit (large *N*) at critical point. The divergence of two point correlation makes the autocorrelation of the magnetization time series divergent. Thus the strong multifractality of the Ising model around critical point should at least caused in part by the increase of autocorrelation.

**Fig 8:**
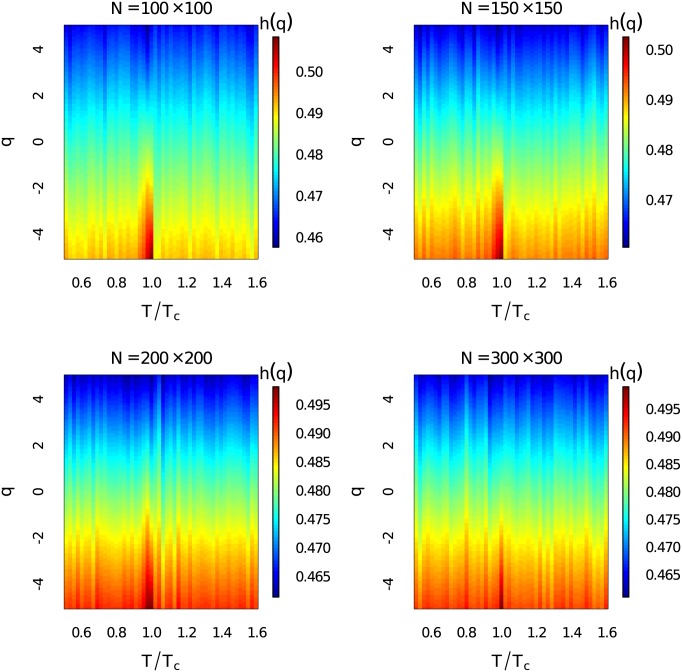
Generalized Hurst exponent for shuffled time series. (Color online) Heat map of ensemble average of the generalized Hurst exponents *h*(*q*) for *q* ∈ [−5, 5] at different temperatures for the shuffled time series with different system sizes.

**Fig 9:**
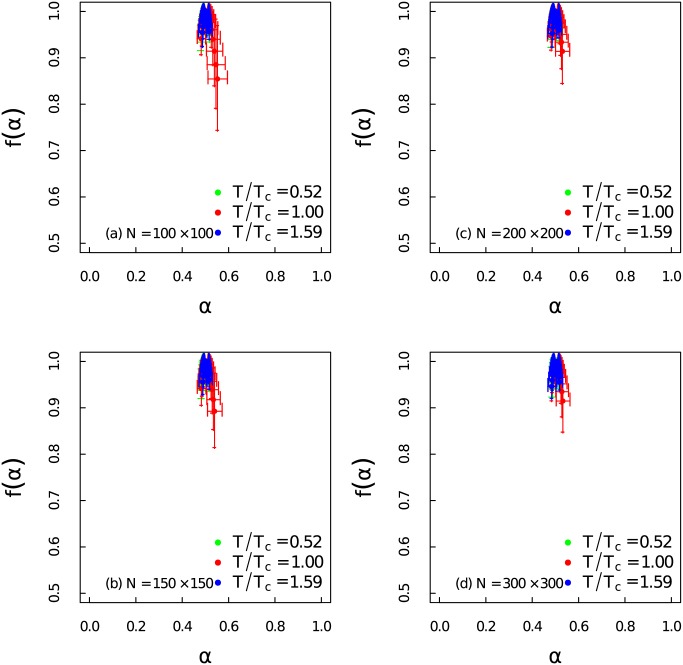
Singularity spectrum for shuffled time series. (Color online) Singularity spectrum *f*(*α*) of the shuffled time series as a function of the singularity strength *α* at different temperatures for different system sizes.

There are two main sources of multifracality which we would like to distinguish [50, 51]: (i) Multifractality due to broad probability density function. (ii) Multifractality due to different long-term correlations of small and large fluctuations. Here we carry out the same analysis as in Figs 3 and 6, but shuffle the data randomly to better identify the sources of multifractality. Multifractality caused by broad probability density function can not be fully eliminated by the shuffling procedure. However, multifractality induced by long-term correlations can be removed after shuffling the time series. The shuffling procedures have been performed 1,000 × *l* transpositions on each time series with 100 ensemble average. While *l* = 100,000 is the length of each time series. Fig 8 gives the dependency between *h*(*q*) and *q* for the shuffled time series at different temperatures with different system sizes. The non-linear dependency only exists near the critical point. In Fig 9 we show that the singularity spectrum of shuffled time series at three temperature regions for different system sizes. We find that the singularity spectrum at *T*/*T*_*c*_ = 0.52 and *T*/*T*_*c*_ = 1.59 are nearly overlapped at one point with *α*_0_ ≃ 0.5. However, the singularity spectrum of the time series near critical point still posses multifractality with a right skewed shape. We address that the sources of strong multifractality of the system near critical region stem from both long-term correlations and broad probability density function [53].

### Visibility Graph

We use the visibility graph method to convert magnetization time series at different temperatures to complex networks. From each 10,000 element-long time series, a network with 10,000 nodes is generated using the visibility graph method. We should mind that the data length used here for visibility graph analysis is only 10,000. This is significantly shorter than what we use in MF-DFA. This is due to the intrinsic data consuming property of the MF-DFA. Then different topological quantities [12] have been calculated. Those topological quantities can characterize the geometrical properties of time series which are directly related to the dynamics of the Ising model.

**Fig 10:**
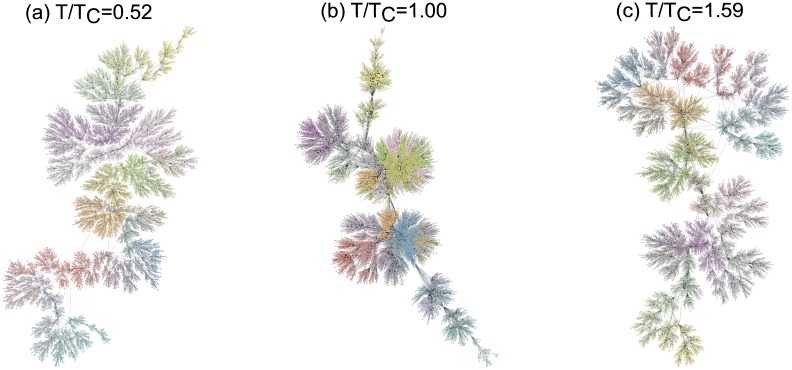
Visibility graphs at different temperatures. (Color online) The network structures of the visibility graphs at (a) *T*/*T*_*c*_ = 0.52, (b) *T*/*T*_*c*_ = 1.00, (c) *T*/*T*_*c*_ = 1.59 for system size *N* = 100 × 100. The network sizes are 10,000. Different colors represent different communities.

Here we give three networks obtained via visibility graph method from time series at three different temperatures in Fig 10. Fig 10(a)–10(c) are the visibility graphs for *T*/*T*_*c*_ = 0.52, 1.00 and 1.59 respectively. The network at *T*/*T*_*c*_ = 1.00 is markedly different from two networks at *T*/*T*_*c*_ = 0.52 and 1.59. The extreme modular network structure is exhibited in Fig 10(b). It can be understood that when *T*/*T*_*c*_ = 1.00 the large trends of the time series make some extreme values have massive visibilities. This is also responsible for the formation of large communities presented by different colors. Two networks in Fig 10(a) and 10(c) possess tree-like structures. The community sizes of these two networks are smaller than that of the network at *T*/*T*_*c*_ = 1.00. The lack of trends leads to the snowflake shapes. Thus the geometric outlines of the time series at different temperatures are preserved due to the affine invariant features of the visibility graph method.

**Fig 11:**
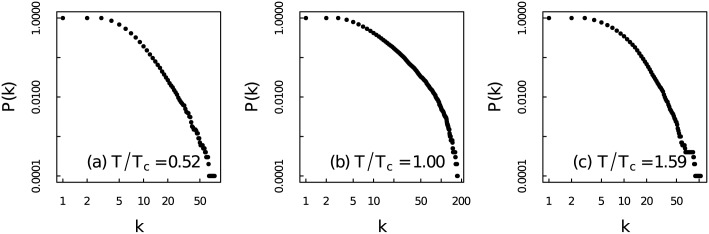
Cumulative degree distributions of the visibility graphs. Cumulative degree distributions of the networks at three different temperatures with (a) *T*/*T*_*c*_ = 0.52, (b) *T*/*T*_*c*_ = 1.00, (c) *T*/*T*_*c*_ = 1.59 for system size *N* = 100 × 100.

**Fig 12:**
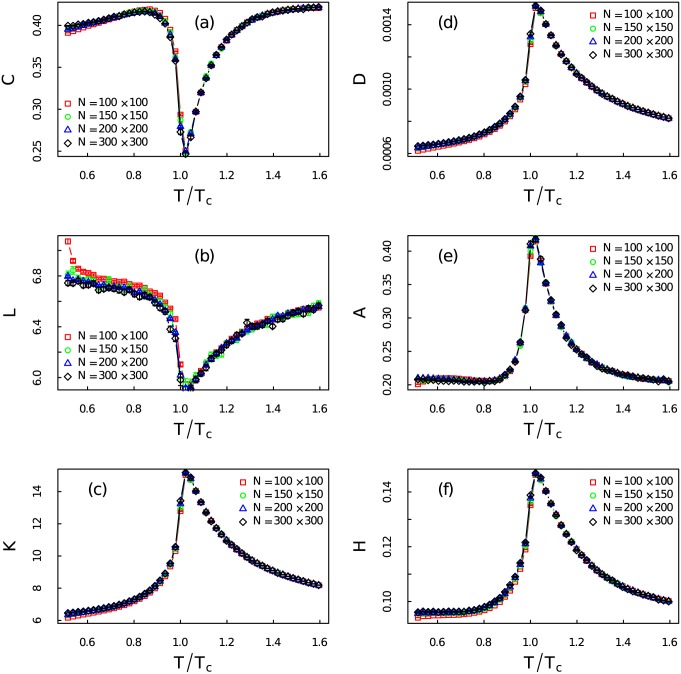
Topological quantities of the visibility graphs. (Color online) Ensemble average of the topological quantities (a) the clustering coefficient *C*, (b) the average degree *K*, (c) the average shortest-path length *L*, (d) the density *D*, (e) the assortativity *A*, (f) the heterogeneity *H* of networks converted from time series at different temperatures. Different colors and symbols represent different system sizes.

Fig 11(a)–11(c) are the degree distributions of networks for *T*/*T*_*c*_ = 0.52, *T*/*T*_*c*_ = 1.00 and *T*/*T*_*c*_ = 1.59, respectively. Three distributions show the heterogeneous nature of the networks. The network at *T*/*T*_*c*_ = 1.00 with broader degree distribution is apparently more heterogeneous than those networks at *T*/*T*_*c*_ = 0.52 and *T*/*T*_*c*_ = 1.59. The largest degree of the network at *T*/*T*_*c*_ = 1.00 is 288 which is significantly larger than the largest degree of those networks away from critical point. According to Ref [14], fractal time series can be converted to scale-free networks. We have tested the scale-free properties of those networks by the method proposed by Clauset and Newman [54]. It turns out that none of them is strictly scale-free network. But they do share heterogeneous nature. Thus we use the heterogeneity index [55] to quantify the degree distribution of those networks. In Fig 12(f): heterogeneity index of the networks increases dramatically near critical point. Hence from the degree distributions of networks we can gain some insights about the fractal nature of magnetization time series which has been discussed by using MF-DFA.

Networks at different regions share the heterogeneous nature, but they do have unique identities which are related to the dynamical properties of the Ising model at specific temperatures. In Fig 12, we calculate the ensemble average of the topological quantities at different temperatures. In Fig 12(a), the clustering coefficient *C*, and (b) the average shortest-path length *L* both decrease around the critical point. Decreases of *C* and *L* around critical point indicate more heterogeneous network structures. We can verify these changes from Fig 12(f). According to Ref [55], heterogeneity of the BA network [43] is around 0.11 which is much smaller than that of the network at critical point. The extreme heterogeneous network structures near critical region are due to the utmost non-stationarity and long-term correlations of the time series which are exactly the characterizations of critical state of the the Ising model. The average degree *K*, and the network density *D* in Fig 12(c) and 12(d) reach their maxima around critical threshold. The network becomes more and more dense when the system approaches *T*_*c*_. Fig 12(e) shows the assortativity *A* as a function of relative temperature. The rapid increase of *A* around *T*_*c*_ gives a hint about the coming of the critical threshold. It also tells that the network becomes more and more assortative which means the existence of long trends and extreme values in the time series near *T*_*c*_. All those topological transitions suggest vast structure distinctions between networks at different temperature regions. Those results have shown geometrical structure transitions of the time series are signals of phase transition from the view of complex networks. The way of those topological quantities approach the critical threshold either form the low or high temperatures manifest the possibility of been used as early-warnings.

As a final remark of the paper, we shall emphasize the importance of our previous results from the view of early-warnings [1, 46, 56]. Recently, plenty of early-warnings have been proposed and summarized [1, 46, 48, 57–64]. Those early-warnings can be categorized into two major classes: metric-based indicators which probe the delicate changes in the statistical properties of the time series, and model-based indicators which detect the changes in the time series dynamics fitted by reasonable models. Thus our results shown in previous sections are metric-based estimators. The moments given in Fig 2 has been fully discussed in Ref [46]. It is exactly consistent with previous studies that the critical slowing down and the fluctuation patterns of the complex system near critical thresholds will increase the autocorrelation, variance and skewness [65–67]. The contribution of our work is that the MF-DFA and visibility graph elaborate the multifractal and geometrical properties of the Ising model near critical point in the time domain. To our best knowledge, this is the first time to use the variation of the multifractality and network properties as early-warnings. In fact due to the divergence of the spatial correlation of the Ising model near critical threshold, the spatial multifractal features may also be used as early-warnings. We can use the higher dimensional MF-DFA [68–70] to explore this problem. This should be subject to the future investigations.

## Conclusions

In conclusion, we have used the multifractal detrended analysis (MF-DFA) and the visibility graph method to analyze the outputs of the two-dimensional Ising model—magnetization time series. Dynamics of the system at different temperatures are directly related to the multifractal and geometrical properties of time series. First four order statistical moments confirmed the existence of phase transition around theoretical critical temperature. The variance, skewness and kurtosis have been shown as three very efficient early-warnings as discussed in recent Ref [46]. According to the MF-DFA, classical Hurst exponent *h*(*q* = 2) shows the extreme non-stationarity of magnetization time series near critical temperature. The generalized Hurst exponents uncover the transformation of time series form weak multifractal (or monofractal) to strong multifractal when temperature approaches critical region. The singularity spectrum and the complexity parameters have been employed to inspect multifractality level of magnetization time series at different temperatures. The shape of singularity spectrum around critical point becomes very complicated and the evolution of the complexity parameters manifest the strong multifractality of the system at critical region quantitatively. The shuffling procedure has identified the sources of multifractality of the system near critical point stem from both broad probability density function and long-term correlations. Meanwhile the visibility graph method has been employed to convert the magnetization time series to complex networks. Heterogeneous degree distributions of the complex networks at three temperature regions have shown the fractal nature of magnetization time series. Basic topological quantities of networks can capture the geometrical variation of time series. The decreases and increases of those topological quantities near critical region have unfolded the critical dynamics of the Ising model.

The evolution patterns of the multifractality and the geometrical properties of the visibility graphs can help us identifying how far the system is away from critical point. Thus we then conclude that the level of multifractality and the topological quantities of visibility graphs can serve as early-warnings for diverse of complex systems. We may notice that the visibility graph method only use the data sets that are 10% of the length used for MF-DFA. This indicates that the visibility graph method maybe more suitable for real world systems since data sets obtained from real world systems are not always very long. To sum up, the MF-DFA and the visibility graph method may not only be limited here for the analysis of the two-dimensional Ising model, but can be used as powerful tools to explore the critical behaviours of many other models and real systems such as Langevin model and financial index [62].
